# Ventilation of Hospitals

**Published:** 1866-01

**Authors:** 


### Ventilation of Hospitals.

Dr. Gallard of La Pitiè, who has lately paid much attention to the ventilation of hospitals, has arrived at the conclusion that *natural* is far superior to *artificial* ventilation. At Hospital Beaujon, he says, has been in operation since 1856 Van Hecke's system of ventilation in the female surgical pavilion of that hospital; and yet statistics show that the mortality has, if anything, increased. In 1851, there was one death to 17.16 patients treated; in 1852, one to 16.26; in 1853, one to 14.48; in 1854, one to 13.81; in 1855, one to 16.23; in 1856, one to 14.25; in 1857, one to 14.93; in 1858, one to 12.85; in 1859, one to 17.14; in 1860, one to 12; in 1861, one to 11.60; in 1862, one to 12.30. At Hospital Necker, where the same apparatus is is operation, the mortality has remained precisely as it previously was, viz: one in eight or nine patients. Then, again, we find still more striking results if we compare the mortality of La Pitié, where there is no artificial ventilation, with that of Lariboisière, where the most expensive system prevails. Lariboisière nominally contains 606 beds, but its average number is 634. La Pitié contains 620. Lariboisière is placed on a height, and stands in an open space. La Pitié is placed low; has muddy waters, tanneries, etc., around it; its superficial area is 21.7 *metres.* Lariboisière has 51.8 *metres;* its beds have each 52 to 53 cubic *metres*, whilst those of La Pitié have from 25 to 49 only. These and other circumstances place La Pitié in a hygienic position greatly inferior to that of Lariboisière. And we should naturally be led to conclude that the mortality of La Pitié was greatest; but such was not the case. We find from statistical comparison of the mortality at the two hospitals since 1858, when Lariboisière was opened, that the mortality in both was much the same. During eleven years, Lariboisière has received 100,718 patients, of whom 12,616, or one in 7.9 died; whilst La Pitié received 103,707, of whom 13,189, or one in 7.8 died. Facts like these, M. Gallard contends, show that artificial systems of ventilation of hospitals are, to say the least of them, useless. Then, again, it appears that during these years, in which statistics were taken, the average duration of diseases in Lariboisière is about two days longer than in La Pitié. It is needless to add, that the expense attending artificial ventilation is great. M. Gallard has often noticed an inconveniently high temperature in wards artificially ventilated. In Lariboisière, where the ventilator of Farcot is employed, the temperature is generally about 17° to 18° cent., and sometimes even above 20°, when 15° or 16° should have been the maximum. The original expenditure on the ventilating apparatus at Lariboisière was upwards of £16,000; and every year the expense of heating the hospital is more than £3,000, whilst that of La Pitié costs only £1,000.—*Brit. Med. Journ.*, Sept. 23, 1865.—*Med. News.*

